# Decellularized small intestine submucosa device for temporomandibular joint meniscus repair: Acute timepoint safety study

**DOI:** 10.1371/journal.pone.0273336

**Published:** 2022-08-25

**Authors:** William L. Chung, Bryan N. Brown, Alejandro J. Almarza

**Affiliations:** 1 Oral and Maxillofacial Surgery, University of Pittsburgh, Pittsburgh, Pennsylvania, United States of America; 2 Department of Bioengineering, University of Pittsburgh, Pittsburgh, Pennsylvania, United States of America; 3 McGowan Institute for Regenerative Medicine, University of Pittsburgh, Pittsburgh, Pennsylvania, United States of America; 4 Department of Oral and Craniofacial Sciences, University of Pittsburgh, Pittsburgh, Pennsylvania, United States of America; 5 Center for Craniofacial Regeneration, University of Pittsburgh, Pittsburgh, Pennsylvania, United States of America; University College London Institute of Child Health, UNITED KINGDOM

## Abstract

Temporomandibular joint (TMJ) Meniscus removal is an option for the patient to regain full range of motion if the disc is irreversibly damaged or unable to be reduced. However, this procedure leaves the joint vulnerable to condylar remodeling and degeneration. We have shown that extracellular matrix (ECM) scaffolds remodel into a tissue with near native TMJ meniscus in previous studies. The next step towards clinical translation is to manufacture the ECM scaffold as a device under good manufacturing practices (GMP) and test it in a pre-clinical animal study under good laboratory practices (GLP). The primary objective of this study was to evaluate the in-vivo histopathological response to a Prototype GMP manufactured device made of decellularized porcine small intestinal submucosa (SIS), by observing for signs of surrounding tissue reaction to the device that are indicative of an adverse host response in comparison to an empty control at 21 days post-surgical implantation in a canine TMJ meniscus removal and implant model in a GLP setting. The conclusive findings were that the ECM device is safe for placement in the TMJ. After 21 days post implantation, histology of tissue surrounding the device and draining lymph nodes showed that the Prototype GMP device had no negative effects compared to the empty site (as evaluated by the board-certified veterinary pathologist). Furthermore, there was a lack of negative findings for clinical pathology (hematology and clinical chemistry), mortality, and body weight/weight change. Future studies will go to one year after implantation to show that the remodel device remains as a viable tissue with near native mechanical properties.

## Introduction

For individuals experiencing painful clicking or locking of the temporomandibular joint (TMJ), the condition dramatically impacts the quality of their lives and interferes with normal activities such as talking, yawning, or chewing/mastication of food. Locking of the TMJ meniscus (disc) is also known as TMJ meniscus displacement without reduction (MDwoR) or “closed-lock” of the jaw, and MDwoR is one of the specific conditions under the temporomandibular disorder (TMD) umbrella. As is highlighted in the NASEM Consensus Report, TMD often occurs with comorbid conditions including fatigue, widespread pain, fibromyalgia, depression, anxiety, and arthritic conditions [1]. Furthermore, the report states that it is not unusual for multiple diagnoses attributed to TMD to be present in the same individual. Such overlap can make it difficult to distinguish which specific diagnosis is primary or which is necessarily the best target for treatment [1]. Conservative self-management remains one of few treatment modalities that is supported by clinical evidence for improved outcomes. However, surgery remains a critical component of TMD care for properly selected patients [1].

For a percentage of TMD patients, histologic evidence suggests that the posterior attachment of the meniscus may undergo change or remodeling from elastic connective tissue to dense connective tissue or “pseudo meniscus” over time (generally greater than 6 months), restoring masticatory function and motion of the mandible. However, this does not occur in all patients and MDwoR causes substantial pain, limitation of normal activities, and disability until remodeling occurs. Therefore, when MDwoR presents with chronic (>3 months) closed lock, and the patient does not improve maximal mouth opening (MMO) after months of conservative treatment [2], the patient and clinician may decide to remove the meniscus (meniscectomy) [3]. It should be noted that there is insufficient evidence to show that surgical management for MDwoR with or without closed-lock improves either pain or MMO when compared to conservative treatments [2, 4–6], indicating the need for more clinical studies. Furthermore, time to regain MMO and quality of life during the intervening period was not considered in those studies and reviews. Meniscectomy increases MMO immediately; however, this procedure also leaves the joint vulnerable to condylar remodeling and degeneration, often leading to the need for additional surgery [7–9], which is why an alternative solution is needed.

It is estimated that the incidence of patients with MDwoR is 2% to 8% of patients that seek treatment for TMDs [10, 11]. It is impossible to predict how many of those patients will opt for a meniscectomy after conservative treatments if MMO has not improved to a functional state, and the literature on MDwoR is not clear on how many patients with symptoms decide on surgical intervention [12–24]. Nevertheless, regeneration of the TMJ meniscus will provide an alternative to carefully selected patients [25].

Currently, no alloplastic alternatives exist to safely and effectively replace the TMJ meniscus. Previous attempts to use alloplastic materials have resulted in unsatisfactory outcomes, including increased pathology and other complications [26–29]. Some autografts are currently being used, like the temporalis muscle or a dermis/adipose tissue graft, but most resorb within 12 months post-implantation [30–36]. A lot of these patients instead undergo complete removal of the meniscus (meniscectomy) leading to bone-on-bone articulation, which causes osteoarthritic damage, and perhaps the eventual need for a total joint prosthetic. Thus, the identification of an effective off-the-shelf replacement would represent a significant clinical advance, obviating donor site morbidity for autografts and avoiding osteoarthritis of the condyle.

Our approach is to use extracellular matrix (ECM) scaffolds as a template for remodeling of the TMJ meniscus. ECM scaffolds have been shown to be effective biomaterials for support of de novo, site appropriate tissue formation in a wide range of preclinical and clinical studies spanning multiple tissue and organ systems [37, 38]. We have published studies both using decellularized porcine urinary bladder (UBM) in a canine model [39, 40], and canine small intestine submucosa (SIS) in a porcine model [41]. Both models showed that cell-free ECM scaffolds are an effective template material for reconstruction of the TMJ meniscus following a clinically relevant meniscectomy procedure. In these studies, a device consisting of a powdered ECM “pillow” encapsulated within sheets of the same material was placed as an interpositional graft after meniscectomy. The implanted material was observed to progressively remodel from 3 weeks to 6 months after implantation, and the newly formed host tissues resembled the native fibrocartilage of the TMJ meniscus in both gross and histologic morphology [40]. In addition, the collagen and GAG content, along with the compression mechanical properties of the remodeled tissue were 100% of that of the native meniscus. In these studies, the tensile properties of the remodeled ECM scaffold achieved greater than 50% of native TMJ meniscus tissue [41]. Of note, placement of the device resulted in formation of fibrocartilage within the bulk of the implant and site-appropriate muscular and ligamentous attachments at the periphery of the implant.

The next step towards clinical translation is to manufacture the ECM scaffold as a device under good manufacturing practices (GMP) and test it in a pre-clinical animal study under good laboratory practices (GLP). The primary objective of this study was to evaluate the in-vivo histopathological response of a Prototype GMP device made of porcine SIS, by observing for signs of surrounding tissue reaction to the device that are indicative of a distinct host response in comparison to an empty control, at 22 days post-surgical implantation in a canine TMJ (temporomandibular joint) meniscus implant model in a GLP setting. The secondary objectives were to observe for negative impacts in clinical pathology, mortality, and body weight/weight change. Thus, there are two novel aspects to this work: 1) This is the first time we use porcine SIS in a canine TMJ model; and 2) this is the first time a prototype GMP ECM scaffold is used in an GLP animal study.

## Materials and methods

### Device specifications

The device manufacture was done under “Prototype GMP” conditions, which mean very carefully and under controlled conditions and with well-documented processes and instructions. Only after a device goes though very rigorous design controls, regulatory approval, transfer to manufacturing, and a host of validations and audits can it truly be considered manufactured under GMP. The device is composed solely of decellularized porcine small intestine submucosa (SIS) extracellular matrix without the addition of any chemical stabilizers or crosslinking agent, or other chemical compound. The device is made from 4-layers of SIS hydrated sheets with 150–200 mg of SIS powder in between the second and third SIS sheet making a pillow of dried powder in the center of the device [39–41]. The SIS powder is lyophilized, and remaining portion of the device is vacuum pressed. The device is packaged in double-pouched (one inner, one outer) in two polyethylene Tyvek pouches for sterilization and as primary device packaging. Ethylene Oxide was used to sterilize the device, and then it was stored at room temperature.

### Animal welfare

All animal work was conducted at an AAALAC accredited Testing Facility (Charles River Laboratories, Mattawan, MI). This study complied with all applicable sections of the Final Rules of the Animal Welfare Act regulations (Code of Federal Regulations, Title 9), the *Public Health Service Policy on Humane Care and Use of Laboratory Animals* from the Office of Laboratory Animal Welfare, and the *Guide for the Care and Use of Laboratory Animals* from the National Research Council. The protocol and any amendments or procedures involving the care or use of animals in this study were reviewed and approved by the Testing Facility’s Institutional Animal Care and Use Committee before the initiation of such procedures.

### Surgeries

**Fig 1:**
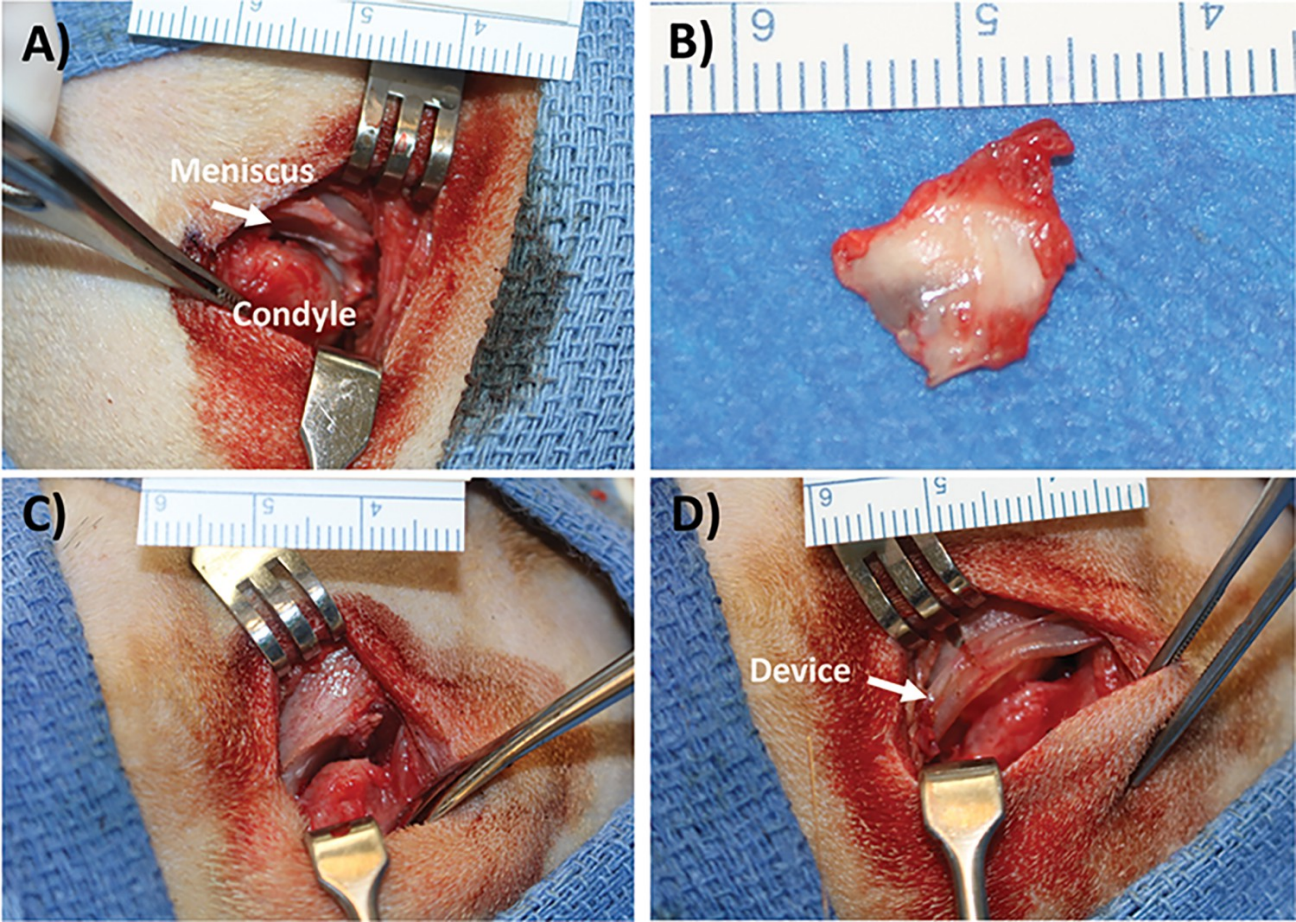
Representative images of meniscus removal and device placement. (A) Joint is opened and meniscus is identified. (B) Meniscus is removed. Note: The whole meniscus is often not removed at once, but in this sample most of the disc was isolated. Medial remnants sometimes remain. (C) The joint is then empty. (D) Device is placed in joint space and lip is curled around fossa with suture through drilled holes.

Five skeletally mature (12 to 15 months of age) female beagles, weighing between 6.1 and 7.2 kg were used for this study (Marshall BioResources, North Rose, NY). Animals were initially pair housed prior to study start and individually housed post-surgery. All the dogs were sedated with acepromazine maleate (0.1mg/kg body weight) before intubation and maintained in a surgical plane of anesthesia with isoflurane (to effect). A full listing of preoperative, surgery and postoperative medications are maintained by the Testing Facility and included in the final report. The surgical site was shaved and then prepared using a chlorohexidine scrub before the placement of sterile drapes. An incision was performed anterior to the tragus, preserving the local innervation and vasculature. The native meniscus was exposed and then completely excised (Fig 1). The same meniscus removal procedure was performed on both experimental and control sides. After excision of the native meniscus on the experimental side, the meniscus was replaced with the ECM device as described in the next paragraph. The contralateral side did not receive an implant.

The ECM device was hydrated in sterile saline (0.9%) for approximately 10 minutes before implantation as a replacement device for the native meniscus. The implants were inserted such that the powder “pillow” is positioned between the temporal fossa and the condylar head (Fig 1). Three holes were created in the temporal fossa, and the implants were secured to the temporal fossa using slow resorbing fixation sutures. Fixation sutures were be placed in the anterior and posterior aspects of the implant to adjacent soft tissue. All fixation sutures were placed through the sheet portion of the ECM device. The skin was closed using resorbable suture material.

### Postoperative care

Following the ECM device surgery implant procedure, the animals were recovered from anesthesia, extubated, and monitored until resting comfortably in a sternal position. The animals received post operative monitoring for clinical observations and implantation site evaluations and post operative medications (Meloxicam SR, and Skintegrity), and food and environmental enrichments were provided at the discretion of the veterinarian staff as needed. Animals were provided a soft diet for the first 7 days postoperatively and were returned to a normal laboratory diet thereafter.

Body weight was measured at the time of acceptance into the study, prior to surgery, twice weekly for the duration of the study, and prior to necropsy.

### MRI imaging

Animals were anesthetized with acepromazine maleate (0.1 mg/kg, SC), atropine sulfate (0.05 mg/kg, SC), propofol (6.0 mg/kg, IV) and isoflurane (to effect, inhalation) for MRI imaging. T2 weighted images were taken to visualize if the joint space collapses from resorption of the ECM device, or the maintenance of the remodeled device. MRI was performed on both joints (device and empty) right after implantation (Day 1) and right before necropsy (Day 22±1). Animals were scanned in dorsal recumbency with a 1.5T Phillips Intera, MRI scanner with PowerTrac 6000 gradients (Phillips Healthcare) located at the Testing Facility. Images were obtained using the C4 linear surface RF coil. All scans were performed closed-mouth with the following parameters; Coronal views were taken utilizing an FOV: 90 mm, pixel size of 0.4 x 0.4 mm; 2.0 mm slice, and TR = 2261; TE = 30; NSA = 3. Sagittal views were taken utilizing an FOV: 90 mm, pixel size of 0.3 x 0.5 mm; 2.0 mm slice, and TR = 1716; TE = 20; NSA = 4.

### Hematology and clinical chemistry

Blood was collected at the time of surgery and shortly before necropsy from the jugular vein for hematology and clinical chemistry. Specifically, 1 ml of blood was collected with K_2_EDTA as an anticoagulant for hematology, and 1.8 ml of blood with a Serum Gel Separator for clinical chemistry.

The parameters measured for hematology were: WBC-White Blood Cell Count, NEUT-Neutrophils, LYMPH-Lymphocytes, MONO-Monocytes, EOS-Eosinophils, BASO-Basophils, LUC-Large Unstained Cells, RBC-Red Blood Cell Count, HGB-Hemoglobin, HCT-Hematocrit, MCV-Mean Corpuscular Volume, MCH-Mean Corpuscular Hemoglobin, MCHC-Mean Corpuscular Hemoglobin Concentration, RDW-Red Blood Cell Distribution Width, PLT-Platelet Count, and RETIC-Reticulocytes.

The parameters measured for clinical chemistry were: AST-Aspartate Aminotransferase, ALT-Alanine Aminotransferase, ALP-Alkaline Phosphatase, GGT-Gamma Glutamyl Transferase, TBIL-Total Bilirubin, UREAN-Urea Nitrogen, CREAT-Creatinine, GLUC-Glucose, CHOL-Cholesterol, TRIG-Triglycerides, TPROT-Total Protein, ALB-Albumin, GLOB-Globulin, A/G-Albumin/Globulin Ratio, CA-Calcium, PHOS-Phosphorus, NA-Sodium, K-Potassium, CL-Chloride, Hemolysis Indice, Lipemia Indice, Icterus Indice.

### Euthanasia

On Day 22, the animals were euthanized by intravenous administration of an approved euthanasia solution (sodium pentobarbital based). Post-mortem, the temporal fossa condylar head, and interpositional material between the structures were excised en-bloc.

### Histology

The entire implant site was removed en-bloc including a portion of the mandibular condyle, temporal fossa, joint space, graft material (right side only) and surrounding tissue. The samples were placed in 10% neutral buffered formalin (NBF) and shipped to the test site to process each joint as an en-block tissue.

Prior to decalcification, radiographs of the tissue were taken to aid in trimming. The joints were decalcified (using a solution optimized for immunohistochemistry) and trimmed along a sagittal plane to produce up to four tissue slabs per joint. Each of the resulting slabs were processed and embedded in paraffin and sectioned at ~5–8 μm. Up to five serial sections were taken from each of two levels in each block, with the levels spaced 120 μm apart (10 total sections per block, up to 40 sections per joint). One section was stained with H&E from each of the levels (8 sections per joint) for the treated and untreated joints.

Additionally, a single section from each of the treated joints was immunolabeled [implant site] with MAC387 monoclonal antibody (macrophages). Prior to performing immunohistochemistry, a pathologist reviewed the H&E sections to identify the best levels for staining in each treated sample.

Tissues were evaluated microscopically by a board-certified veterinary pathologist. The following scale was used: NP = Not Present; N = Normal; 1 = Minimal; 2 = Mild; 3 = Moderate; 4 = Marked; 5 = Severe.

### Regulatory compliance

The study was performed in accordance with the U.S. Department of Health and Human Services, Food and Drug Administration, United States Code of Federal Regulations, Title 21, Part 58: Good Laboratory Practice for Nonclinical Laboratory Studies and as accepted by Regulatory Authorities throughout the European Union (OECD Principles of Good Laboratory Practice), Japan (MHLW), and other countries that are signatories to the OECD Mutual Acceptance of Data Agreement.

Exceptions to GLPs include the following study elements: Characterization of the device was performed by the Cook Biotech Inc. at a laboratory that follows FDA Good Manufacturing Practice (GMP) regulations; The MRI system has not been validated per FDA guidance 21CFR Part 11.

The design of this study was based on the study objectives, the overall product development strategy for the test article, and the following study design guidelines: ISO 10993–6: 2016 –Biological evaluation of medical devices–Part 6: Test for local effect after implantation.

## Results

### Surgical procedures

Surgeries were performed as planned on study. An unforeseen complication was that, while implanting the ECM device in some animals, the ECM device was larger than what could be comfortably accommodated within the joint space. As a result, the ECM device could not be easily placed within the joint space and required additional effort for insertion, which resulted in the device slipping out of the joint space in two out of the five animals. Ease of insertion varied from animal to animal.

### Detailed clinical observations

Swelling of the face and mouth was noted in all animals on study and varied from light to severe in nature. These observations are consistent with the recovery from the procedure but were more prominent on the side receiving the ECM device. Decreased activity, and abnormal consistency of feces were also noted, but were largely attributed to the surgical procedure and modified, soft diet.

### Body weight and body weight gains

On average, mean body weights were within a normal range for this species, sex, breed and age (low: 5.98 kg (Day 22) to high: 6.73 kg (Day -2). On average animals experienced a minor loss in weight over the course of the study (-0.75 kg). This could be largely attributable to the surgical procedure itself and the modified diet received during recovery.

### Hematology

There were no implant-related effects among hematology parameters in all animals (Tables 1–3). All fluctuations among individual and mean values, regardless of statistical significance, were considered sporadic, consistent with biologic variation and/or negligible in magnitude, and not procedural related.

**Table 1:** Hematology reporting for each animal for WBC, NEUT, LYMPH, MONO, EOS, and BASO.

		WBC	NEUT	LYMPH	MONO	EOS	BASO
	Day(s) Relative to Start Date	(10^3/uL)	(10^3/uL)	(10^3/uL)	(10^3/uL)	(10^3/uL)	(10^3/uL)
1001	-6	19.56	14.61	3.31	1.24	0.21	0.12
	21	8.67	5.5	2.54	0.43	0.13	0.04
1002	-6	9.37	5.07	3.44	0.43	0.29	0.07
	21	7.55	4.36	2.65	0.26	0.18	0.06
1003	-7	10.27	5.64	4.07	0.32	0.12	0.08
	21	8.36	4.76	2.94	0.46	0.07	0.07
1004	-7	8.61	5.36	2.56	0.39	0.16	0.1
	21	7.21	3.8	2.8	0.3	0.22	0.05
1005	-7	9.3	5.68	2.91	0.38	0.21	0.06
	21	6.15	3.04	2.68	0.15	0.13	0.09

**Table 2:** Hematology reporting for each animal for LUC, RBC, HGB, HCT, MCV, and MCH.

		LUC	RBC	HGB	HCT	MCV	MCH
	Day(s) Relative to Start Date	(10^3/uL)	(10^6/uL)	(g/dL)	(%)	(fL)	(pg)
1001	-6	0.07	8.04	18.5	54.4	67.6	23
	21	0.04	7.94	18.1	54.1	68.2	22.8
1002	-6	0.08	6.63	15.5	45.5	68.7	23.4
	21	0.04	7.08	16.6	49	69.2	23.5
1003	-7	0.05	7.74	17	50.4	65.1	22
	21	0.05	8.39	18.1	53.5	63.8	21.6
1004	-7	0.05	7.11	16.5	48.3	67.9	23.2
	21	0.04	7.93	18.3	53.3	67.2	23.1
1005	-7	0.06	6.53	15	43.8	67.1	23
	21	0.07	8.36	18.8	56	67	22.5

**Table 3:** Hematology reporting for each animal for MCHC, RDW, PLT, and RETIC.

		MCHC	RDW	PLT	RETIC
	Day(s) Relative to Start Date	(g/dL)	(%)	(10^3/uL)	(10^9/L)
1001	-6	34	13.1	316	87.5
	21	33.4	13.2	365	52
1002	-6	34	12.8	304	45.1
	21	34	12.8	338	44.9
1003	-7	33.8	12.6	257	61.6
	21	33.8	13.1	262	81.4
1004	-7	34.2	12.3	284	23.1
	21	34.4	13	315	29.5
1005	-7	34.3	12.3	378	47.8
	21	33.6	12.8	631	57.4

### Clinical chemistry

There were no implant-related effects among clinical chemistry parameters in all animals (Tables 4–7). All fluctuations among individual and mean values were considered sporadic, consistent with biologic variation and/or negligible in magnitude, and not related to ECM device administration.

**Table 4:** Biochemistry reporting for each animal for AST, ALT, ALP, GGT, TBIL, and UREAN.

		AST	ALT	ALP	GGT	TBIL	UREAN
	Day(s) Relative to Start Date	(U/L)	(U/L)	(U/L)	(U/L)	(mg/dL)	(mg/dL)
1001	-6	26	25	49	5	0.14	12
	21	20	14	24	3	0.13	15
1002	-6	22	22	56	4	0.15	22
	21	22	22	25	3	0.17	15
1003	-7	21	29	33	3	0.37	16
	21	21	18	28	3	0.12	12
1004	-7	29	25	41	4	0.22	18
	21	28	19	23	3	0.19	19
1005	-7	28	39	74	4	0.19	16
	21	35	39	26	4	0.2	17

**Table 5:** Biochemistry reporting for each animal for CREAT, GLUC, CHOL, TRIG, TPROT, and ALB.

		CREAT	GLUC	CHOL	TRIG	TPROT	ALB
	Day(s) Relative to Start Date	(mg/dL)	(mg/dL)	(mg/dL)	(mg/dL)	(g/dL)	(g/dL)
1001	-6	0.6	103	136	40	5.5	3
	21	0.6	99	192	37	5.5	3.2
1002	-6	0.8	92	177	34	5.5	2.9
	21	0.6	83	148	33	5.4	3.2
1003	-7	0.5	93	168	57	5.8	3.2
	21	0.5	102	156	42	5.9	3.3
1004	-7	0.6	91	131	35	5.7	3.2
	21	0.7	85	185	48	5.8	3.4
1005	-7	0.7	99	180	31	5.2	2.8
	21	0.7	109	232	41	5.9	3.3

**Table 6:** Biochemistry reporting for each animal for GLOB, A/G, CA, PHOS, NA, K.

		GLOB	A/G	CA	PHOS	NA	K
	Day(s) Relative to Start Date	(g/dL)	(ratio)	(mg/dL)	(mg/dL)	(mEq/L)	(mEq/L)
1001	-6	2.5	1.2	9.8	4.5	145	4.6
	21	2.3	1.4	9.8	4.2	147	4.8
1002	-6	2.6	1.1	10.1	4.9	149	4.8
	21	2.2	1.5	9.6	3.8	148	4.8
1003	-7	2.6	1.2	10.5	4.5	145	4.7
	21	2.6	1.3	10.3	3.7	146	4.6
1004	-7	2.5	1.3	10.1	4.2	146	4.5
	21	2.4	1.4	10.7	3.9	149	4.7
1005	-7	2.4	1.2	9.8	4.8	148	4.4
	21	2.6	1.3	9.8	3.7	148	5.6

**Table 7:** Biochemistry reporting for each animal for CL, Hemolysis Indice, Lipemia Indice, and Icterus Indice.

	Day(s) Relative to Start Date	CL	Hemolysis	Lipemia	Icterus
		(mEq/L)	Indice	Indice	Indice
1001	-6	111	N	N	N
	21	112	N	N	N
1002	-6	114	N	N	N
	21	115	N	N	N
1003	-7	109	N	N	N
	21	111	N	N	N
1004	-7	112	N	N	N
	21	114	N	N	N
1005	-7	114	N	N	N
	21	112	N	N	N

### Draining lymph nodes

The microscopic findings in the draining lymph nodes (mandibular) were similar in the nodes draining the treated (right) and empty control (left) sites. Findings included increase numbers of histiocytes and/or neutrophils, as well as extracellular and cytoplasmic pigment suggestive of hemoglobin break down products. These microscopic findings were considered to be secondary to the surgery and healing processes. There was no clear treatment-related effect present in the lymph nodes.

### Overall joint evaluation (MRI, histology, and necropsy observations)

This is an observational study to assess the safety of the ECM device on five animals. Thus, it is necessary to show these results for each individual animal. Below, findings for each animal are summarized with MRI, histology, and then necropsy findings, for both empty and implanted joints.

#### Animal 1001

**Fig 2:**
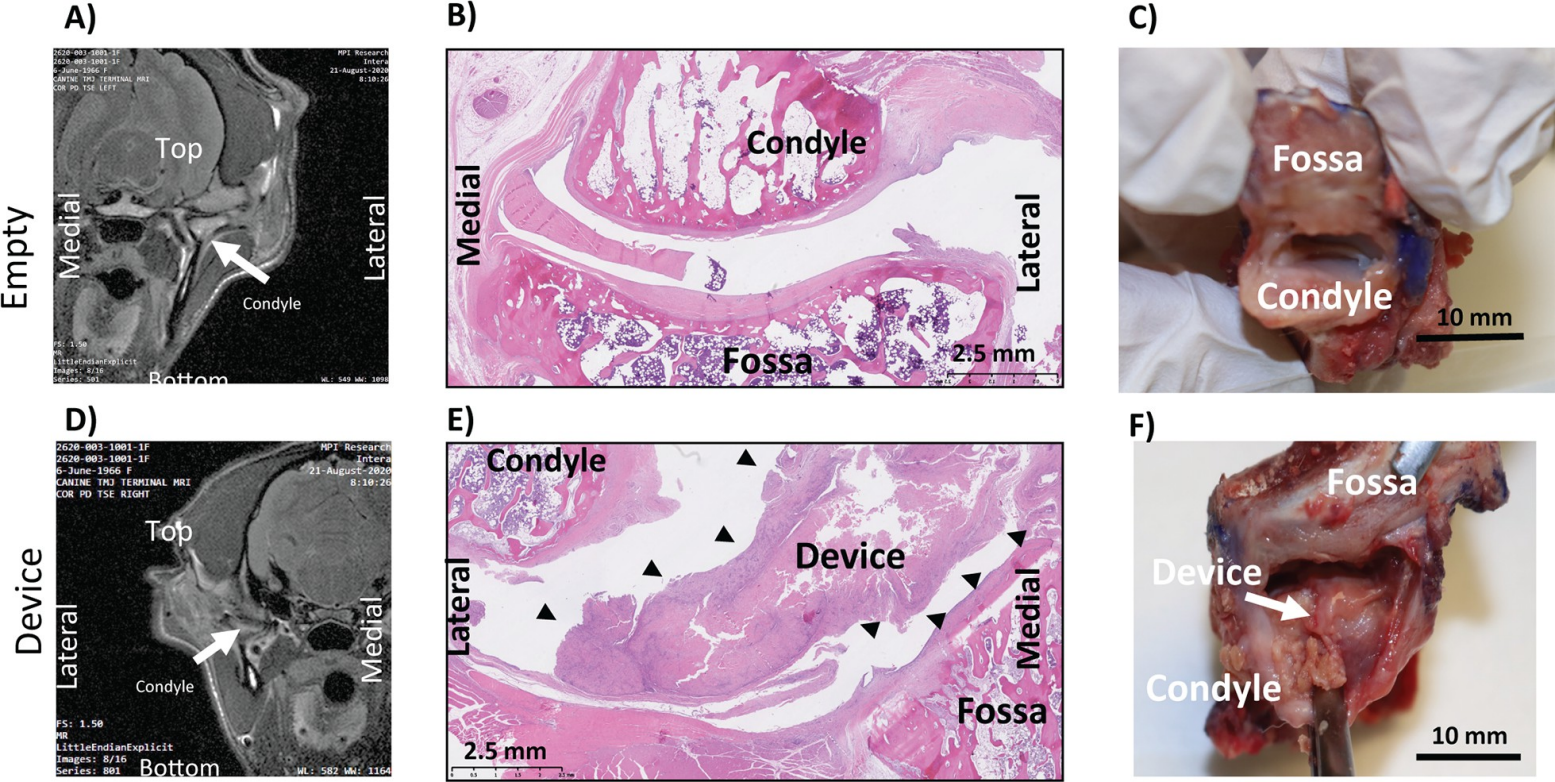
Animal 1001: Coronal MRI of empty (A) and implanted (D), Sagittal H&E of empty (B) and implanted (E), Necropsy Picture of empty (C) and implanted (F).

Empty joint: As seen in Fig 2A, the MRI shows the joint space collapsed. The histology in Fig 2B shows no new meniscus regeneration and some remnant meniscus. Fig 2C shows dissection of joint with opened joint capsule, and clearly showing no tissue on top of condyle surface.

Implanted joint: As seen in Fig 2D for the MRI, it seems like there is tissue separating the condyle from the fossa. The histology in Fig 2E shows the ECM device in the joint space. Furthermore, the surface and superficial portions of the ECM device material were infiltrated by mixed immune cells (macrophages, lymphocytes, rare neutrophils and giant cells) while the center of the ECM device material was generally intact, with little-to-no central cellular infiltration. Fig 2F shows dissection of joint with opened joint capsule, and device is clearly seen in joint space. Histopathological scores were similar between implanted and empty joints (Table 8).

**Table 8:** Individual histopathological findings for animal 1001.

	Animal No. 1001																					
	Day 22±1																					
	Right TMJ (Treated)											Left TMJ (empty control)										
	Slide 1	Slide 2	Slide 3	Slide 4	Slide 5	Slide 6	Slide 7	Slide 8	INC			Slide 1	Slide 2	Slide 3	Slide 4	Slide 5	Slide 6	Slide 7	Slide 8	INC		
Bone-resorption at joint surface	NP	NP	NP	NP	3	3	1	NP	3	/	8	NP	NP	2	3	NP	NP	NP	NP	2	/	8
Bone—new periosteal formation	NP	NP	NP	NP	1	1	1	NP	3	/	8	NP	NP	1	1	NP	NP	NP	NP	2	/	8
Articular surface—fibrous	NP	NP	NP	NP	2	2	NP	NP	2	/	8	NP	NP	1	1	4	4	NP	NP	4	/	8
Articular surface—fibrovascular	NP	NP	NP	NP	3	3	1	NP	3	/	8	NP	NP	3	3	1	1	NP	NP	4	/	8
Articular surface—cartilage	NP	NP	NP	NP	NP	NP	4	NP	1	/	8	NP	NP	2	2	NP	NP	NP	NP	2	/	8
Tissue/bare	NP	NP	NP	NP	NP	NP	NP	NP	0	/	8	NP	NP	NP	NP	NP	NP	NP	NP	0	/	8
Joint space—article present	NP	NP	NP	NP	5	5	NP	NP	2	/	8	NP	NP	NP	NP	NP	NP	NP	NP	0	/	8
Joint space—fibrovascular tissue present	NP	NP	NP	NP	1	1	NP	NP	2	/	8	NP	NP	2	2	NP	NP	NP	NP	2	/	8
Joint space—fibrous tissue present	NP	NP	NP	NP	1	1	1	NP	3	/	8	NP	NP	NP	NP	2	2	NP	NP	2	/	8
Joint space—tissue debris present	NP	NP	NP	NP	NP	NP	1	NP	1	/	8	NP	NP	NP	NP	NP	NP	NP	NP	0	/	8
Joint periphery—immune cell infiltration	NP	NP	NP	NP	NP	NP	NP	NP	0	/	8	NP	NP	NP	NP	NP	1L	NP	NP	1	/	8
Article—total amount remaining	5	5	5	5	3	3	NP	3	7	/	8	NP	NP	NP	NP	NP	NP	NP	NP	0	/	8
Article—immune cell response within article	3	3	3	3	4	4	NP	4	7	/	8	NP	NP	NP	NP	NP	NP	NP	NP	0	/	8
Presence of sutures and inflammatory reaction	1	NP	NP	NP	3	3	NP	NP	2	/	8	NP	NP	NP	NP	NP	NP	NP	NP	0	/	8
Other tissue responses	NP	NP	NP	NP	NP	NP	NP	NP	0	/	8	NP	NP	NP	NP	NP	NP	NP	NP	0	/	8

Key: INC = Incidence; NP = Not Present; N = Normal; 1 = Minimal; 2 = Mild; 3 = Moderate; 4 = Marked; 5 = Severe

#—cartilage present within joint space, score of 3; L = Lymphocyte (number indicated severity); N = Neutrophil (number indicated severity); G = Giant cell (number indicated severity); T = Thrombus within vessel adjacent to the joint; recanalization present; TI = Thrombus within vessels adjacent to joint with complete disruption of the wall and mixed inflammation (vasculitis); LA = lymphocyte aggregation near article; H = Hair shafts or other foreign material with peripheral mixed inflammation; M = Skeletal muscle adjacent to the joint infiltrated by lymphocytes; NF = Fibrosis within nerve adjacent to joint

Note: On the H&E-stained sections, the level of macrophage infiltration in the joint periphery appeared to be similar in all sites—this evaluation factor was not scored Note: the synovium in the joint periphery of all joints had minimal to no proliferative responses; this evaluation factor was not scored

Note: immune cells were not freely present within any joint space—this evaluation factor was not scored

#### Animal 1002

**Fig 3:**
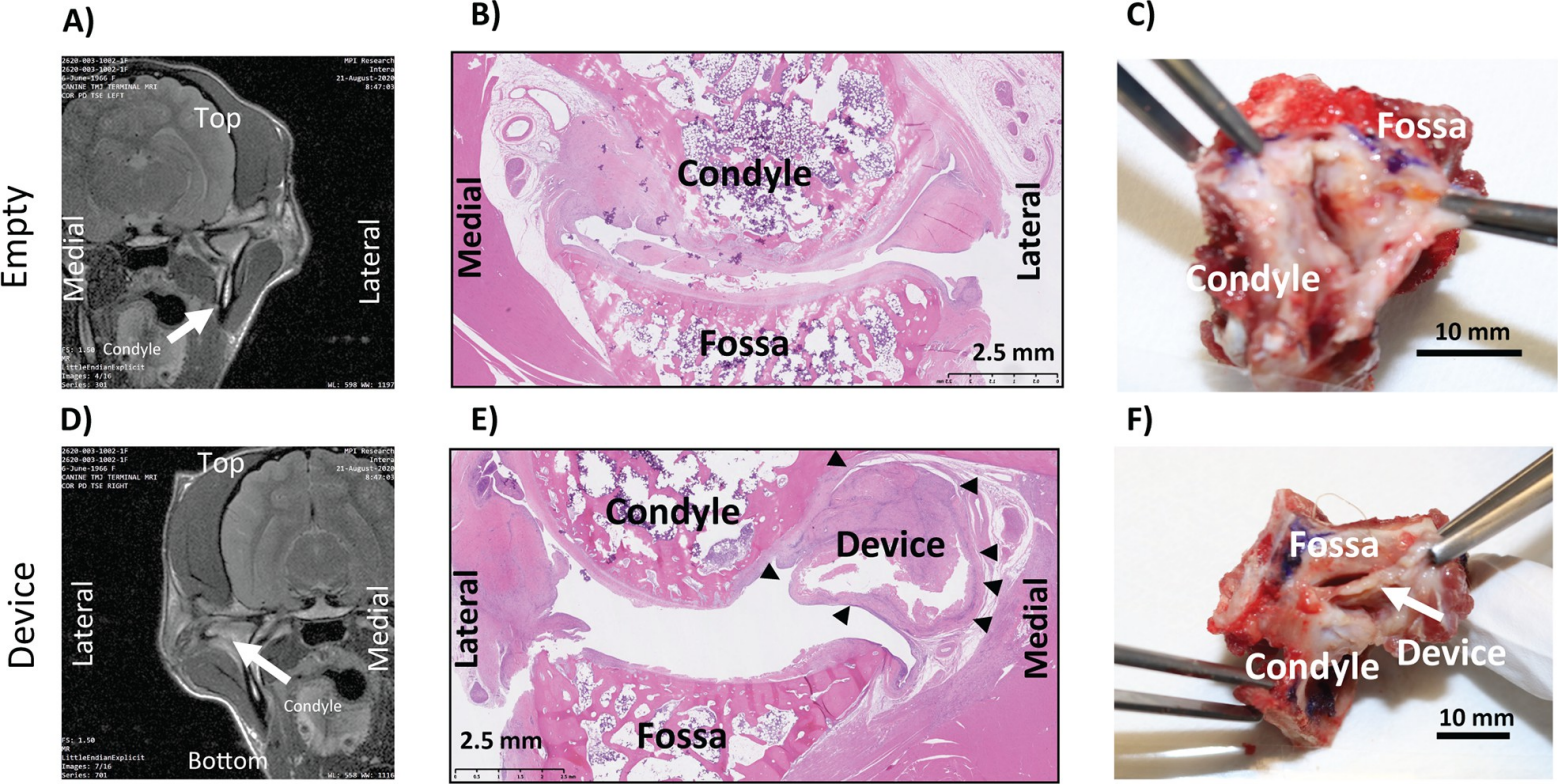
Animal 1002: Coronal MRI of empty (A) and implanted (D), Sagittal H&E of empty (B) and implanted (E), Necropsy Picture of empty (C) and implanted (F).

Empty joint: As seen in Fig 3A, the MRI shows the joint space collapsed. The histology in Fig 3B shows no new meniscus regeneration. Fig 3C shows dissection of joint with opened joint capsule, and no tissue was observed on top of condyle surface.

Implanted joint: As seen in Fig 3D for the MRI, it is clear that there is tissue separating the condyle from the fossa. The histology in Fig 3E shows the device in the joint space, but not in the central region of the articular surface, and cellular infiltration throughout the device. The inflammatory response in the ECM device-treated joints was primarily present within and immediately adjacent to EMC device. Inflammatory responses distant to the ECM device were similar to the responses seen in empty control joints. Since the cellular response associated with remaining ECM device material was primarily macrophages and/or lymphocytes, this increased cellular response was interpreted as being primarily a resorptive response (host removing article from the implant site) and not a specific immune response to the ECM device itself. Fig 3F shows dissection of joint with opened joint capsule, and device is clearly seen in joint space. Histopathological scores were similar between implanted and empty joints (Table 9).

**Table 9:** Individual histopathological findings for animal 1002.

	Animal No. 1002																					
	Day 22±1																					
	Right TMJ (Treated)											Left TMJ (empty control)										
	Slide 1	Slide 2	Slide 3	Slide 4	Slide 5	Slide 6	Slide 7	Slide 8	INC			Slide 1	Slide 2	Slide 3	Slide 4	Slide 5	Slide 6	Slide 7	Slide 8	INC		
Bone-resorption at joint surface	NP	NP	NP	NP	2	3	1	1	4	/	8	2	2	3	3	2	NP	NP	NP	5	/	8
Bone—new periosteal formation	NP	NP	NP	NP	1	1	1	1	4	/	8	1	1	1	1	1	1	1	1	8	/	8
Articular surface—fibrous	NP	NP	NP	NP	2	1	NP	NP	2	/	8	NP	NP	NP	1	1	1	4	4	5	/	8
Articular surface—fibrovascular	NP	NP	NP	NP	2	3	1	1	4	/	8	5	5	5	4	2	2	1	1	8	/	8
Articular surface—cartilage	NP	NP	NP	NP	NP	NP	3	2	2	/	8	NP	NP	NP	NP	2	2	NP	NP	2	/	8
Tissue/bare	NP	NP	NP	NP	1	1	1	2	4	/	8	NP	NP	NP	NP	NP	NP	NP	NP	0	/	8
Joint space—article present	NP	NP	NP	NP	2	1	1	1	4	/	8	NP	NP	NP	NP	NP	NP	NP	NP	0	/	8
Joint space—fibrovascular tissue present	NP	NP	NP	NP	3	3	2	1	4	/	8	NP	1	NP	NP	1	1	1	1	5	/	8
Joint space—fibrous tissue present	NP	NP	NP	NP	2	2	NP	NP	2	/	8	NP	NP	NP	NP	3	2	5	5	4	/	8
Joint space—tissue debris present	NP	NP	NP	NP	1	1	1	1	4	/	8	1	NP	1	1	1	1	NP	NP	5	/	8
Joint periphery—immune cell infiltration	NP	NP	NP	NP	2L	1L	3L	3L	4	/	8	NP	NP	NP	1L	NP	NP	NP	NP	1	/	8
Article—total amount remaining	4	4	4	4	3	2	2	2	8	/	8	NP	NP	NP	NP	NP	NP	NP	NP	0	/	8
Article—immune cell response within article	4	4	4	4	4	3	2	2	8	/	8	NP	NP	NP	NP	NP	NP	NP	NP	0	/	8
Presence of sutures and inflammatory reaction	2	2	3	3	2	2	NP	NP	6	/	8	NP	NP	NP	NP	NP	NP	NP	NP	0	/	8
Other tissue responses	NP	NP	NP	NP	NP	NP	M	M	0	/	8	NP	NP	NP	NP	NP	NP	NP	NP	0	/	8

Key: INC = Incidence; NP = Not Present; N = Normal; 1 = Minimal; 2 = Mild; 3 = Moderate; 4 = Marked; 5 = Severe

#—cartilage present within joint space, score of 3; L = Lymphocyte (number indicated severity); N = Neutrophil (number indicated severity); G = Giant cell (number indicated severity); T = Thrombus within vessel adjacent to the joint; recanalization present; TI = Thrombus within vessels adjacent to joint with complete disruption of the wall and mixed inflammation (vasculitis); LA = lymphocyte aggregation near article; H = Hair shafts or other foreign material with peripheral mixed inflammation; M = Skeletal muscle adjacent to the joint infiltrated by lymphocytes; NF = Fibrosis within nerve adjacent to joint

Note: On the H&E-stained sections, the level of macrophage infiltration in the joint periphery appeared to be similar in all sites—this evaluation factor was not scored Note: the synovium in the joint periphery of all joints had minimal to no proliferative responses; this evaluation factor was not scored

Note: immune cells were not freely present within any joint space—this evaluation factor was not scored

#### Animal 1003

**Fig 4:**
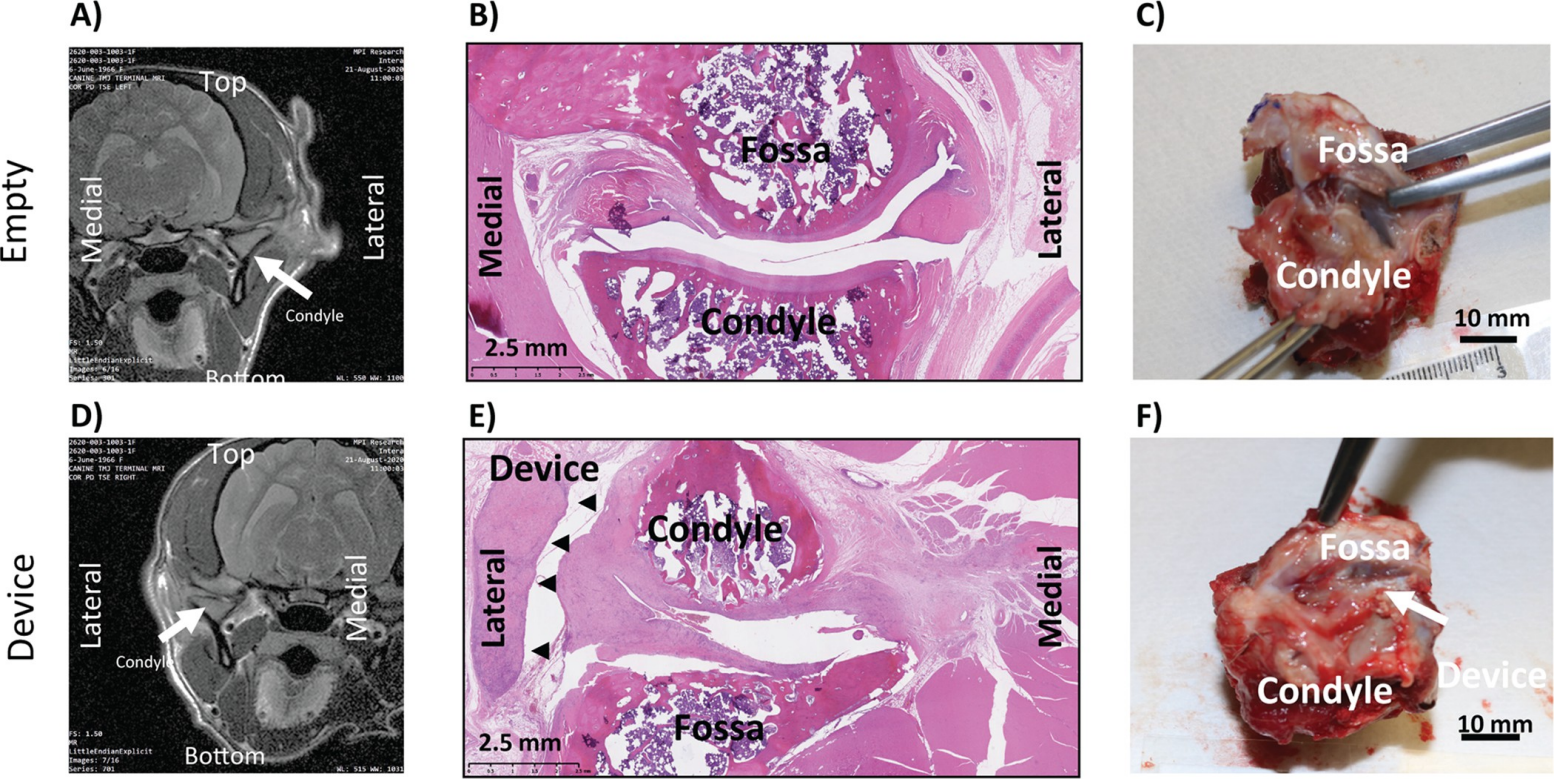
Animal 1003: Coronal MRI of empty (A) and implanted (D), Sagittal H&E of empty (B) and implanted (E), Necropsy Picture of empty (C) and implanted (F).

Empty joint: As seen in Fig 4A, the MRI shows the joint space collapsed. The histology in Fig 4B shows no new meniscus regeneration. Fig 4C shows dissection of joint, but the joint capsule was not opened.

Implanted joint: As seen in Fig 4D for the MRI, it is clear that there is tissue separating the condyle from the fossa. The histology in Fig 4E shows the device adjacent to the joint space (but not between the articular surfaces), and cellular infiltration throughout the device. Microscopic findings in structures adjacent to the TMJ were more common in the empty control (left) joint than in the treated joint. Changes were noted in vessels (thrombosis, inflammation), nerves (fibrosis), and skeletal muscle (lymphocytic infiltration). The reason for the increased incidence of adjacent tissue findings in empty control joints was not apparent microscopically. Fig 4F shows dissection of joint, but the joint capsule was not opened, and the edge of the device appears to be visible. Histopathological scores were similar between implanted and empty joints (Table 10).

**Table 10:** Individual histopathological findings for animal 1003.

	Animal No. 1003																					
	Day 22±1																					
	Right TMJ (Treated)											Left TMJ (empty control)										
	Slide 1	Slide 2	Slide 3	Slide 4	Slide 5	Slide 6	Slide 7	Slide 8	INC			Slide 1	Slide 2	Slide 3	Slide 4	Slide 5	Slide 6	Slide 7	Slide 8	INC		
Bone-resorption at joint surface	3	2	1	1	2	3	NP	NP	6	/	8	3	3	NP	NP	1	1	NP	NP	4	/	8
Bone—new periosteal formation	1	1	1	1	1	1	NP	NP	6	/	8	1	1	1	1	1	1	NP	NP	6	/	8
Articular surface—fibrous	NP	NP	NP	NP	2	2	NP	NP	6	/	8	1	1	4	4	4	4	NP	NP	6	/	8
Aticular surface—fibrovascular	5	5	1	1	2	2	NP	NP	6	/	8	4	4	1	1	1	1	NP	NP	6	/	8
Articular surface—cartilage	NP	NP	3	2	NP	NP	NP	NP	6	/	8	NP	NP	NP	NP	NP	NP	NP	NP	0	/	8
Tissue/bare	NP	NP	1	2	1	1	NP	NP	4	/	8	NP	NP	NP	NP	NP	NP	NP	NP	0	/	8
Joint space—article present	NP	NP	NP	NP	NP	NP	NP	NP	0	/	8	NP	NP	NP	NP	NP	NP	NP	NP	0	/	8
Joint space—fibrovascular tissue present	1	1	1	1	1	1	NP	NP	6	/	8	1	1	1	1	1	1	NP	NP	6	/	8
Joint space—fibrous tissue present	NP	NP	NP	NP	4	4	NP	NP	6	/	8	NP	NP	1	1	5	5	NP	NP	4	/	8
Joint space—tissue debris present	2	1	1	1	NP	NP	NP	NP	4	/	8	1	1	NP	NP	NP	NP	NP	NP	2	/	8
Joint periphery—immune cell infiltration	NP	NP	NP	NP	NP	NP	NP	NP	0	/	8	NP	NP	NP	NP	1N	1N	2LP	2LP	4	/	8
Article—total amount remaining	2	2	NP	NP	NP	NP	NP	NP	6	/	8	NP	NP	NP	NP	NP	NP	NP	NP	0	/	8
Article—immune cell response within article	1	1	NP	NP	NP	NP	NP	NP	6	/	8	NP	NP	NP	NP	NP	NP	NP	NP	0	/	8
Presence of sutures and inflammatory reaction	NP	NP	NP	NP	NP	NP	NP	NP	0	/	8	NP	NP	NP	NP	NP	NP	NP	NP	0	/	8
Other tissue responses	NP	NP	NP	NP	NP	NP	NP	NP	0	/	8	T	T	NP	NP	NP	NP	NP	NP	0	/	8

Key: INC = Incidence; NP = Not Present; N = Normal; 1 = Minimal; 2 = Mild; 3 = Moderate; 4 = Marked; 5 = Severe

#—cartilage present within joint space, score of 3; L = Lymphocyte (number indicated severity); N = Neutrophil (number indicated severity); G = Giant cell (number indicated severity); T = Thrombus within vessel adjacent to the joint; recanalization present; TI = Thrombus within vessels adjacent to joint with complete disruption of the wall and mixed inflammation (vasculitis); LA = lymphocyte aggregation near article; H = Hair shafts or other foreign material with peripheral mixed inflammation; M = Skeletal muscle adjacent to the joint infiltrated by lymphocytes; NF = Fibrosis within nerve adjacent to joint

Note: On the H&E-stained sections, the level of macrophage infiltration in the joint periphery appeared to be similar in all sites—this evaluation factor was not scored Note: the synovium in the joint periphery of all joints had minimal to no proliferative responses; this evaluation factor was not scored

Note: immune cells were not freely present within any joint space—this evaluation factor was not scored

#### Animal 1004

**Fig 5:**
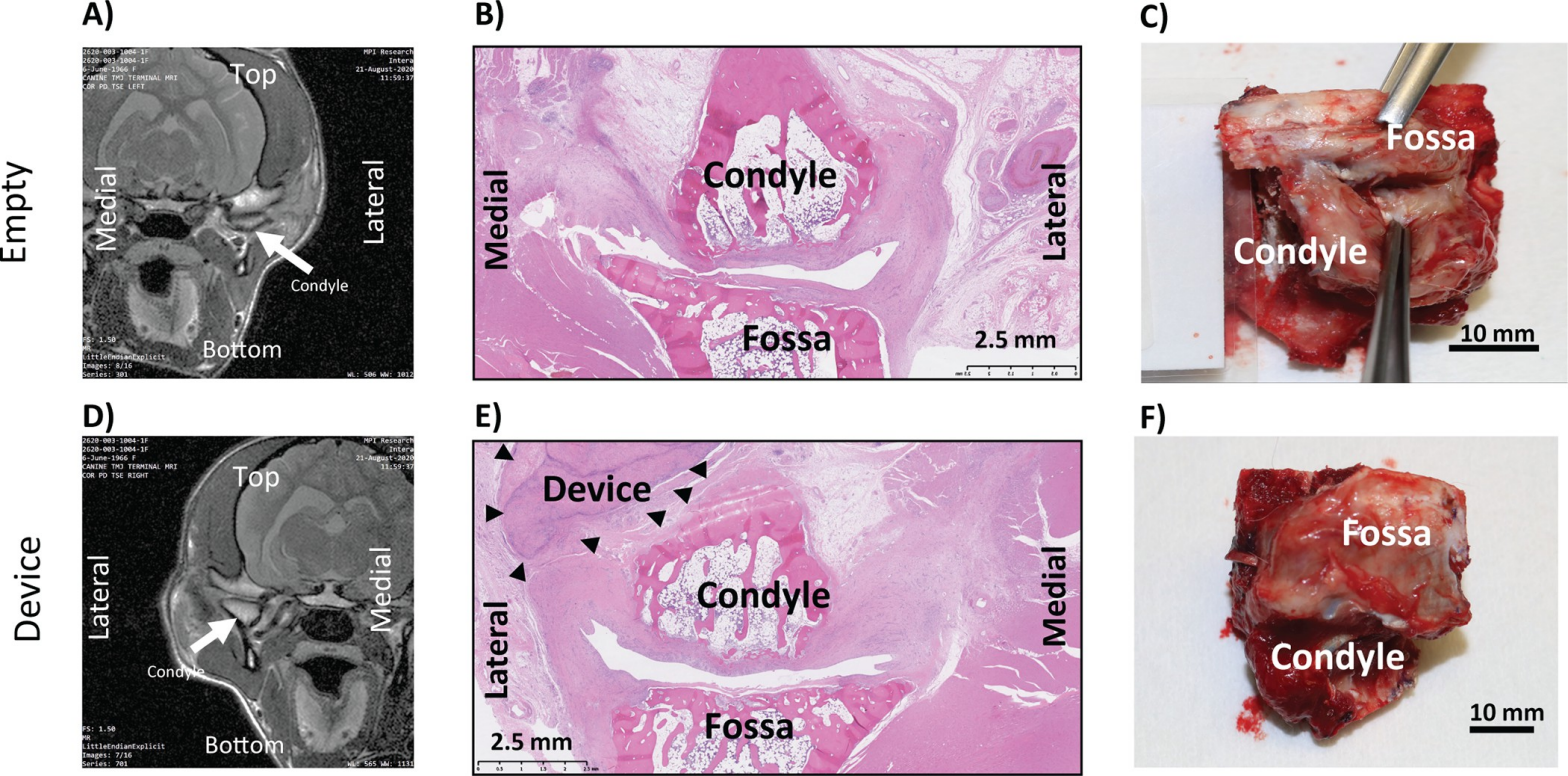
Animal 1004: Coronal MRI of empty (A) and implanted (D), Sagittal H&E of empty (B) and implanted (E), Necropsy Picture of empty (C) and implanted (F).

Empty joint: As seen in Fig 5A, the MRI shows the joint space collapsed. The histology in Fig 5B shows no new meniscus regeneration. Fig 5C shows dissection of joint, but the joint capsule was not opened.

Implanted joint: As seen in Fig 5D for the MRI, it is clear that there is tissue separating the condyle from the fossa. The histology in Fig 5E shows the device well outside the joint space, and cellular infiltration throughout the device. Fig 5F shows dissection of joint, but the joint capsule was not opened. Histopathological scores were similar between implanted and empty joints (Table 11).

**Table 11:** Individual histopathological findings for animal 1004.

	Animal No. 1004																					
	Day 22±1																					
	Right TMJ (Treated)											Left TMJ (empty control)										
	Slide 1	Slide 2	Slide 3	Slide 4	Slide 5	Slide 6	Slide 7	Slide 8	INC			Slide 1	Slide 2	Slide 3	Slide 4	Slide 5	Slide 6	Slide 7	Slide 8	INC		
Bone-resorption at joint surface	NP	NP	NP	NP	3	2	2	2	4	/	8	3	3	3	3	1	1	2	2	8	/	8
Bone—new periosteal formation	NP	NP	NP	NP	1	1	1	1	4	/	8	1	1	1	1	1	1	1	1	8	/	8
Articular surface—fibrous	NP	NP	NP	NP	NP	NP	NP	NP	0	/	8	NP	NP	1	1	2	2	4	4	6	/	8
Aticular surface—fibrovascular	NP	NP	NP	NP	3	3	1	1	4	/	8	5	5	3	3	2	2	1	1	8	/	8
Articular surface—cartilage	NP	NP	NP	NP	NP	NP	2	2	2	/	8	NP	NP	NP	NP	NP	NP	NP	NP	0	/	8
Tissue/bare	NP	NP	NP	NP	2	2	2	2	4	/	8	NP	NP	1	1	1	1	NP	NP	4	/	8
Joint space—article present	NP	NP	NP	NP	NP	NP	NP	NP	0	/	8	NP	NP	NP	NP	NP	NP	NP	NP	0	/	8
Joint space—fibrovascular tissue present	NP	NP	NP	NP	1	1	1	1	4	/	8	2	2	1	1	1	1	1	1	8	/	8
Joint space—fibrous tissue present	NP	NP	NP	NP	NP	NP	NP	NP	0	/	8	NP	NP	NP	NP	NP	NP	5	5	2	/	8
Joint space—tissue debris present	NP	NP	NP	NP	2	1	1	1	4	/	8	1	1	2	2	1	1	NP	NP	6	/	8
Joint periphery—immune cell infiltration	NP	NP	NP	NP	NP	NP	NP	NP	0	/	8	NP	NP	1L	1L	NP	NP	NP	1L	3	/	8
Article—total amount remaining	4	4	3	3	2	2	NP	NP	6	/	8	NP	NP	NP	NP	NP	NP	NP	NP	0	/	8
Article—immune cell response within article	2	2	2	2	3	3	NP	NP	6	/	8	NP	NP	NP	NP	NP	NP	NP	NP	0	/	8
Presence of sutures and inflammatory reaction	NP	NP	NP	NP	NP	NP	NP	NP	0	/	8	NP	NP	NP	NP	NP	NP	NP	NP	0	/	8
Other tissue responses	NP	NP	NP	NP	LA	LA	M	M	4	/	8	TI,	TI,	TI,	TI,	NF	NF	NP	NP	6	/	8
												H, NF	H, NF	H,	H,							
														NF, M	NF, M							

Key: INC = Incidence; NP = Not Present; N = Normal; 1 = Minimal; 2 = Mild; 3 = Moderate; 4 = Marked; 5 = Severe

#—cartilage present within joint space, score of 3; L = Lymphocyte (number indicated severity); N = Neutrophil (number indicated severity); G = Giant cell (number indicated severity); T = Thrombus within vessel adjacent to the joint; recanalization present; TI = Thrombus within vessels adjacent to joint with complete disruption of the wall and mixed inflammation (vasculitis); LA = lymphocyte aggregation near article; H = Hair shafts or other foreign material with peripheral mixed inflammation; M = Skeletal muscle adjacent to the joint infiltrated by lymphocytes; NF = Fibrosis within nerve adjacent to joint

Note: On the H&E-stained sections, the level of macrophage infiltration in the joint periphery appeared to be similar in all sites—this evaluation factor was not scored Note: the synovium in the joint periphery of all joints had minimal to no proliferative responses; this evaluation factor was not scored

Note: immune cells were not freely present within any joint space—this evaluation factor was not scored

#### Animal 1005

**Fig 6:**
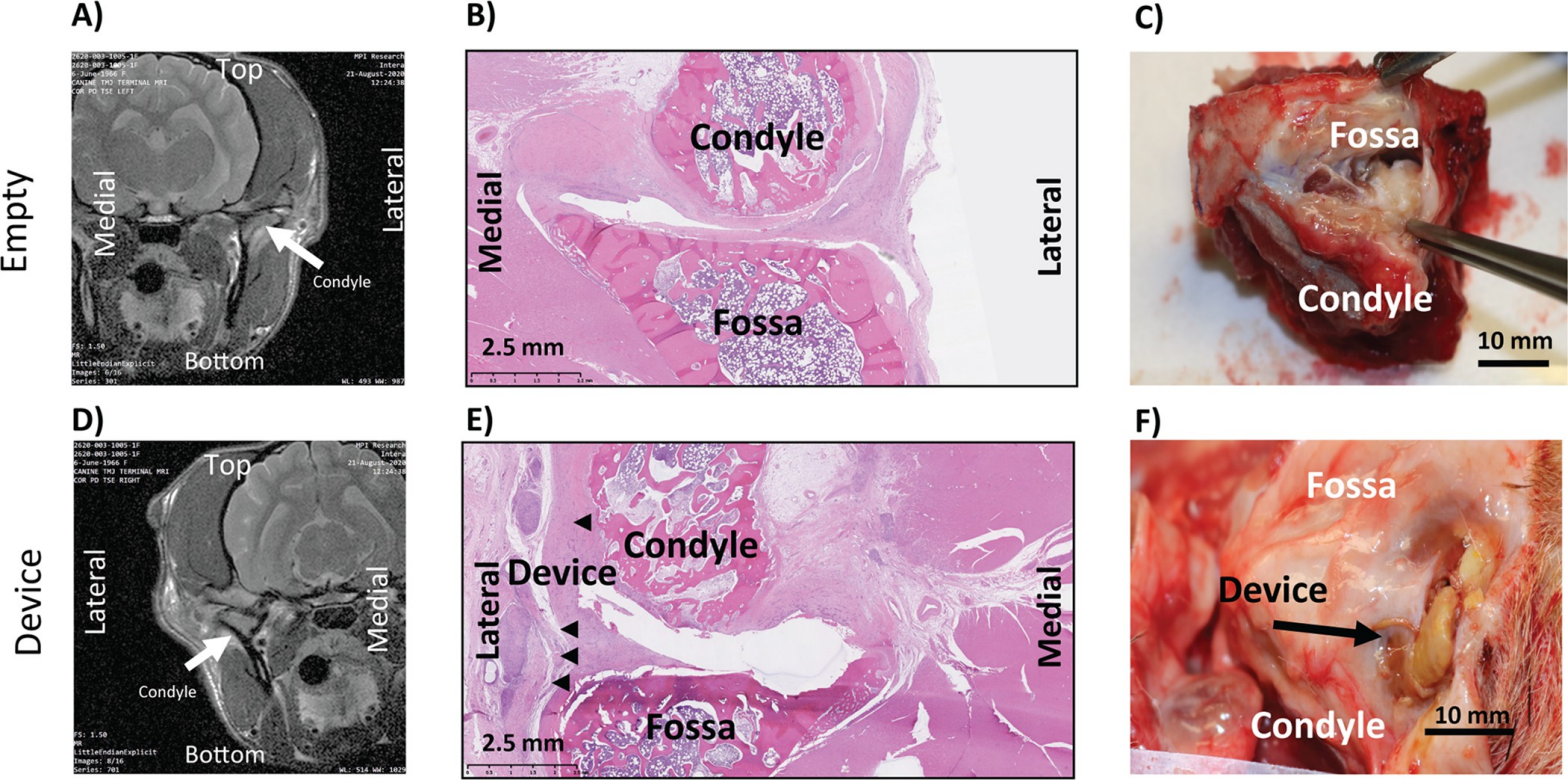
Animal 1005: Coronal MRI of empty (A) and implanted (D), Sagittal H&E of empty (B) and implanted (E), Necropsy Picture of empty (C) and implanted (F).

Empty joint: As seen in Fig 6A, the MRI shows the joint space collapsed. The histology in Fig 6B shows no new meniscus regeneration. Fig 6C shows dissection of joint, but the joint capsule was not opened.

Implanted joint: As seen in Fig 6D for the MRI, it is clear that there is tissue separating the condyle from the fossa. The histology in Fig 6E shows the device well outside the joint space, and cellular infiltration throughout the device. Fig 6F shows dissection of joint, and core of the device could be seen after the muscle was dissected. The implant became separated from the joint space during trimming and imaging. An image was obtained to document this (Fig 6F), and the implant was included with the rest of the sample for histopathology. Histopathological scores were similar between implanted and empty joints (Table 12).

**Table 12:** Individual histopathological findings for animal 1005.

	Animal No. 1005																					
	Day 22±1																					
	Right TMJ (Treated)											Left TMJ (empty control)										
	Slide 1	Slide 2	Slide 3	Slide 4	Slide 5	Slide 6	Slide 7	Slide 8	INC			Slide 1	Slide 2	Slide 3	Slide 4	Slide 5	Slide 6	Slide 7	Slide 8	INC		
Bone-resorption at joint surface	NP	NP	NP	NP	2	2	NP	NP	2	/	8	1	1	1	1	1	1	NP	NP	6	/	8
Bone—new periosteal formation	NP	NP	NP	NP	1	1	1	1	4	/	8	1	1	1	1	1	1	1	1	8	/	8
Articular surface—fibrous	NP	NP	NP	NP	NP	NP	4	4	2	/	8	1	1	1	1	2	2	5	5	8	/	8
Articular surface—fibrovascular	NP	NP	NP	NP	3	2	NP	NP	2	/	8	4	4	2	2	1	1	NP	NP	6	/	8
Articular surface—cartilage	NP	NP	NP	NP	NP	NP	1	1	2	/	8	NP	NP	1	1	1	1	NP	NP	4	/	8
Tissue/bare	NP	NP	NP	NP	2	3	NP	NP	2	/	8	NP	NP	1	1	1	NP	NP	NP	3	/	8
Joint space—article present	NP	NP	NP	NP	NP	NP	NP	NP	0	/	8	NP	NP	NP	NP	NP	NP	NP	NP	0	/	8
Joint space—fibrovascular tissue present	NP	NP	NP	NP	1	1	NP	NP	2	/	8	NP	NP	1	1	1#	1#	1	1	6	/	8
Joint space—fibrous tissue present	NP	NP	NP	NP	NP	NP	5	5	2	/	8	NP	NP	NP	NP	1	1	4	4	4	/	8
Joint space—tissue debris present	NP	NP	NP	NP	1	1	NP	NP	2	/	8	NP	NP	2	2	1	1	1	1	6	/	8
Joint periphery—immune cell infiltration	NP	NP	NP	NP	NP	NP	2L	2L	2	/	8	NP	NP	G, 1	G, 1	NP	NP	NP	NP	2	/	8
Article—total amount remaining	4	4	3	3	1	1	NP	NP	6	/	8	NP	NP	NP	NP	NP	NP	NP	NP	0	/	8
Article—immune cell response within article	4	4	4	4	4	4	NP	NP	8	/	8	NP	NP	NP	NP	NP	NP	NP	NP	0	/	8
Presence of sutures and inflammatory reaction	3	3	3	3	2	2	NP	NP	6	/	8	NP	NP	NP	NP	NP	NP	NP	NP	0	/	8
Other tissue responses	NP	NP	NP	NP	M	M	NP	NP	2	/	8	TI,	TI,	NF, M	NF, M	M	M	NP	NP	6	/	8
												NF, M	NF, M									

Key: INC = Incidence; NP = Not Present; N = Normal; 1 = Minimal; 2 = Mild; 3 = Moderate; 4 = Marked; 5 = Severe

#—cartilage present within joint space, score of 3; L = Lymphocyte (number indicated severity); N = Neutrophil (number indicated severity); G = Giant cell (number indicated severity); T = Thrombus within vessel adjacent to the joint; recanalization present; TI = Thrombus within vessels adjacent to joint with complete disruption of the wall and mixed inflammation (vasculitis); LA = lymphocyte aggregation near article; H = Hair shafts or other foreign material with peripheral mixed inflammation; M = Skeletal muscle adjacent to the joint infiltrated by lymphocytes; NF = Fibrosis within nerve adjacent to joint

Note: On the H&E-stained sections, the level of macrophage infiltration in the joint periphery appeared to be similar in all sites—this evaluation factor was not scored Note: the synovium in the joint periphery of all joints had minimal to no proliferative responses; this evaluation factor was not scored

Note: immune cells were not freely present within any joint space—this evaluation factor was not scored

## Discussion

The conclusive finding of the present study was that the ECM device is safe in the TMJ. After 22 days post implantation, histology of tissue surrounding the device and draining lymph nodes showed that the Prototype GMP device had no negative effects compared to the empty site (as evaluated by the board-certified veterinary pathologist). Furthermore, there was an absence of test article-related findings for clinical pathology (hematology and clinical chemistry), mortality, and body weight/weight change. Both of the base materials, powder and sheets, have FDA clearance, but the configuration of both of them combined into a pillow for the use as a TMJ meniscus replacement is not approved for human use. This study marks the first step towards FDA approval with safety data in a large animal model in an acute timepoint. This study is a pivotal turning point for TMJ regenerative therapies, as it is the first to show that a Prototype GMP device is safe in a GLP preclinical animal study.

While MRI showed the presence of the ECM device in the joint space for all five animals, only two out of five animals had clear evidence of the device within the joint space at the end of histological processing. We attribute this to the mismatch in size of the ECM to the size of the TMJ of the dog breed used. The ECM devices were designed to be similar in size to what will be used in human subjects. These dimensions fit well in our previous studies using the mongrel breed [39, 40] with some room to spare in the joint space. However, the beagle breed from this particular vendor used by the Pre-clinical Contract Research Organization (CRO) was smaller than anticipated. In some animals, the ECM devices were easily inserted into the joint space, but in other animals the fit was tight with little space remaining in the joint. This tight fit might have dislodged the ECM device (Fig 6F) either during the healing period, or during necropsy, en-block isolation, cutting, or general histological processing. We believe that all devices did remain in the joint space for 21 days based on the 21-day MRI images. Nevertheless, moving forward we will use a larger breed and have devices of different sizes to ensure an optimal fit within the joint space.

Unlike our previous studies [39–41], the bulk of the ECM device was observed to remain after 21 days (S1 Fig), but with signs of remodeling in the periphery. We believe that this is due to the ECM device being larger with respect to the size of the joint than in previous studies. We expect the device to keep degrading and then to remodel into a meniscus-like tissue. As the objective of this study was to show the safety of the device in the TMJ, this mismatch in size actually provides a worst-case scenario clinically, with no difference in the host response as compared to empty joints observed. The other differences between this study and our published work is that we used porcine SIS in a canine model, while before we used porcine UBM in a canine model [39, 40] and canine SIS in a porcine model [41]. In the present study, the dogs used were skeletally mature, unlike the pig study [41], which necessitated the use of young, growing animals. This is likely a major contributor for the slower than expected degradation of the ECM device. Nevertheless, the findings of the current study suggest that either SIS or UBM can be used as the base material for the ECM device, regardless of source of species.

As reviewed elsewhere [42], no one animal model resembles the human TMJ in all anatomical areas and function. Nevertheless, the pig has often been described as the “gold-standard” for tissue engineering and regenerative medicine approaches to reconstruction of the TMJ meniscus. However, these assertions are largely based upon post-mortem evaluation. It is important to understand the model-specific advantages and limitations that may exist before embarking into tissue engineering preclinical studies. The advantage of the pig and minipig is that anatomy, physiology, and the properties of the tissues have been well characterized. However, the farm pig has the limitation of continuous growth, which confounds results and makes it not feasible for long-term studies. Also, both in the farm and minipig, the zygomatic arch blocks access into the joint space making the surgical approach to the TMJ meniscus difficult. The advantage of the dog is that the joint space is confined, so in terms of attachment of our interpositional device or ECM bioscaffold, the device is likely to stay in place. While it is true that the dog is a carnivore, and the TMJ is a hinge joint that can only rotate, the type of joint function is not likely to impact the healing potential of the joint. This assertion is supported by our recent studies showing similar remodeling in both canine [39, 40] and porcine [41] models. Furthermore, this ECM technology is a xenogenic implant material, and cross-species implantation is required to evaluate results. Since the majority of FDA approved ECM bioscaffold-based devices are porcine based materials, it is logical to implant porcine based ECM into another species.

Our group is the only one to have published in-vivo large animal work to replace the entire TMJ meniscus. The closest related work is a recent study investigating the use of allogeneic costal chondrocytes for the development of a transplantable, cell-based, scaffold-free TMJ implants [43]. These implants were then placed into partial thickness defects of the TMJ meniscus in a mini-pig model. Implants were found to be well integrated, and the mechanical strength of the defects was more robust in the implant-meniscus than in untreated defects. These findings were associated with reduced observations of pathologic or abnormal condylar remodeling in treated animals and provided one of the first proof-of-concept studies of a cell-based tissue engineered meniscus replacement in an animal model. The main difference is that it was only a small partial focal defect (hole) on the meniscus, and not the entire tissue. Furthermore, as the minipig was used, a complex fenestration procedure had to be performed in order to reach the meniscus without fracturing the zygomatic arch. There is another group that attempted to replace the entire TMJ meniscus in a sheep model, but the Poly(εcaprolactone) devices used either did not degrade or eroded the joint articular surfaces [44–49].

Moving forward, we must show that the device remodels into tissue that is still in the joint space one year after implantation, and that the new tissue has near native mechanical properties. Early evaluations of Teflon and silicone meniscus replacement materials showed failure as early as periods of 12-18months or as long as 10 years or more with gross morphological findings of: “foreign body reaction, synovitis, dystrophic calcification, fibrocartilaginous metaplasia, hyalinization, and scarring” [50]. In addition, it was the disastrous failures of the Teflon implants that led to the Class III classification (highest level) of oversight by the FDA. In terms of non-synthetic and degradable materials, patient studies performed to determine the longevity of autografts have determined with MRI that 50% of the original volume is gone by 6 months [51], and the fat appears to become scar or granulation tissue also by 6 months [52]. There are no prostheses available to replace the TMJ disc, but full joint prostheses are available for end-stage of disease, with a low rate of failures in this patient population [53]. In terms of new technologies, 3D printing is providing control over the local architecture of the scaffold [54], which more closely mimics the native ultrastructure of the disc, but these studies have not been translated to in-vivo trials.

Moving forward to translate to the clinic, going to 12 months post-implantation is a long-term timepoint to ensure that the new tissue will remain in the joint and no adverse events are encountered as those seen with Teflon and silicone. In terms of performance, tensile testing that goes beyond quasistatic, such as cyclic or fatigue testing, will likely need to be performed at this 12-month timepoint to predict whether the new remodeled tissue will continue to endure in the complex loading environment of the TMJ. In the future, the indications and counterindications of patients for this scaffold was summarized recently [25].

In conclusion, translating TMJ meniscus technologies from the benchtop to the clinic is a daunting task. All TMJ devices carry the largest level of oversight, class III, which require full premarket approval (PMA) applications with three rounds of patient studies. However, the TMJ market is small when compared to the dental or orthopedic markets. Thus, only technologies that are simple to manufacture have a chance in bridging this translational chasm for TMJ technologies. We believe that this study shows that the ECM device is safe, and it is a candidate for clinical translation.

## Supporting information

S1 Fig Summary of histopathological findings showing changes from the control side in each animal. (TIF)
