# Supplementary figures and images for: Decellularized small intestine submucosa device for temporomandibular joint meniscus repair: Acute timepoint safety study
Source: PLoS One. 2022 Aug 25;17(8):e0273336. doi: 10.1371/journal.pone.0273336 (PMC9409591; doi:10.1371/journal.pone.0273336)

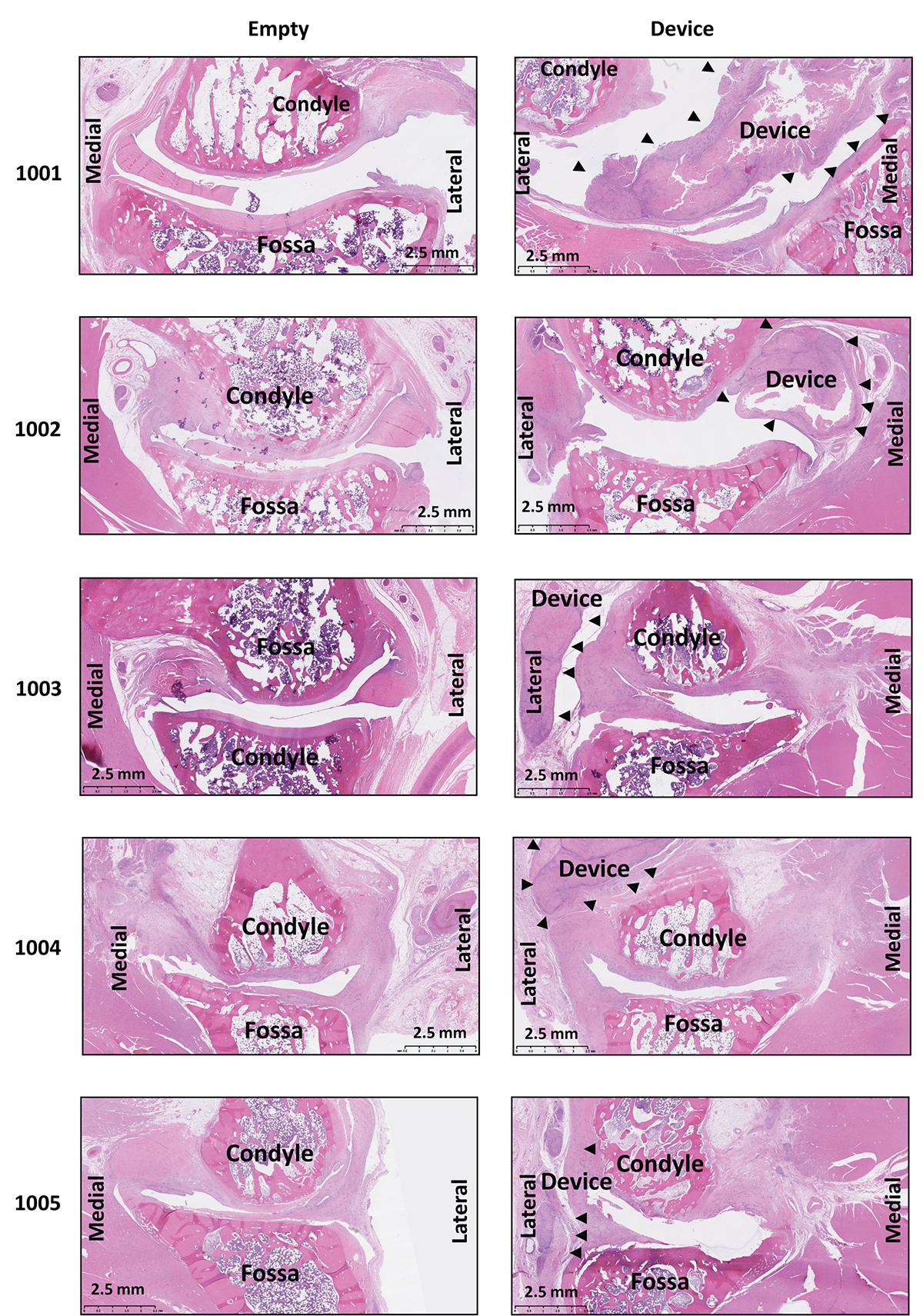

Supplement: S1 Fig — (TIF) [file pone.0273336.s001.tif]
